# Platelet rich plasma compared to viscosupplementation in the treatment of knee osteoarthritis: A systematic review and meta‐analysis of randomised controlled trials with 6 month and 12 month follow‐up

**DOI:** 10.1002/jeo2.70335

**Published:** 2025-07-18

**Authors:** Kian Bagheri, Adithya Shekhar, Eric Kwok, Danton Dungy, Susan L. Stewart, Amir A. Jamali

**Affiliations:** ^1^ Joint Preservation Institute Walnut Creek California USA; ^2^ The Dungy Orthopedic Center Chandler Arizona USA; ^3^ UC Davis School of Medicine Sacramento California USA

**Keywords:** hyaluronic acid, knee, osteoarthritis, PRP, regenerative medicine

## Abstract

**Purpose:**

Platelet rich plasma (PRP) and hyaluronic acid (HA) have been utilised in the last few decades as a conservative treatment for knee osteoarthritis (OA). We sought to evaluate the patient reported outcomes at specific intermediate term endpoints comparing PRP to HA through a systematic review and meta‐analysis of randomised controlled trials (RCTs). We also sought to determine the effect of platelet concentration on the relative outcomes between PRP and HA.

**Methods:**

The Embase, PubMed, Scopus and Cochrane databases were searched for terms related to PRP and osteoarthritis. RCTs comparing PRP and HA in the treatment of knee OA were selected. A total of 26 trials with 1650 knees were included. The two treatments were compared based on the Western Ontario McMaster Universities Osteoarthritis Index (WOMAC) and visual analogue scale (VAS) at specific time points of baseline, 6 months, and 12 months.

**Results:**

PRP had a significant benefit over HA based on the WOMAC at 6 months and at 12 months. It also had a significant benefit over HA on the VAS at 6 months and at 12 months. When limiting the analysis to 6 month follow‐up and separating the studies by platelet count, PRP had a statistically significant benefit over HA for platelet counts corresponding to ‘greater than baseline to 1,250,000 platelets/μL’ for the WOMAC score and platelet counts corresponding to ‘between 750,000 and 1,250,000 platelets/μL’ for the VAS score.

**Conclusions:**

When taken as a whole, PRP demonstrates a significantly superior clinical result compared to HA at 6 months and 12 months. These findings must be considered in light of the numerous preparation protocols and PRP classifications detailed in this report for the included studies.

**Level of Evidence:**

Level 1, systematic review of Level‐1 randomised controlled studies.

**Abbreviations**
ACDA: anticoagulant citrate dextrose solution
BMAC: bone marrow aspirate concentrate
EQ‐VAS: EuroQol Visual Analogue Scale
HA: hyaluronic acid
NSAIDs: non‐steroidal anti‐inflammatory drugs
OA: knee osteoarthritis
PAW: platelets, activation, white cell classification
PRISMA: Preferred Reporting Items for a Systematic Review and Meta‐Analyses
PRO: patient reported outcomes
PRP: platelet rich plasma
RCT: randomised controlled trial
VAS: Visual Analogue Scale
WOMAC: Western Ontario McMaster Universities Osteoarthritis Index

## INTRODUCTION

Knee osteoarthritis (OA) is a degenerative condition characterised by degradation of articular cartilage, synovitis, subchondral bone remodelling, thickening of the joint capsule, formation of osteophytes, ligament degeneration and inflammation [11, 13, 27]. This inflammatory process can be effectively addressed with a number of interventions including oral non‐steroidal anti‐inflammatory drugs (NSAIDs) and intraarticular injections.

Platelet rich plasma (PRP), a concentrate of the blood has also shown promise in effectively controlling this inflammatory process and decreasing pain from knee OA in a number of studies.

Hyaluronic acid (HA) is a macromolecule constituent of the joint synovial fluid. It acts as a normal lubricant and shock absorber in the joint [1]. Exogenous HA administration has been used in the treatment of osteoarthritis. Its mechanism of action has been found to be the direct restoration of lubrication, decrease in synovial inflammation [2, 19, 30, 34, 46] and promotion of endogenous HA production [17, 33].

Studies of PRP for the treatment of knee arthritis have been variable in their study size, degree of knee arthritis among subjects, use of control groups, blinding of patients and investigators/researchers, assignment of treatment, variability of PRP preparation and injection techniques. Many systematic reviews on this topic have only included limited numbers of studies while others have had less rigorous inclusion criteria, often including many non‐randomised series. The literature on PRP reveals numerous variations in technique in preparation of PRP such as volume of blood used, spinning time, spinning speed, number of spins, method of PRP extraction, immediate or delayed injection, number and timing of injections, presence or absence of leucocytes, and use of an activator. In addition to the variation in technique, a plethora of patient reported outcome measures have been utilised in the literature. In order to provide a concise set of findings on the largest groups of subjects possible, we limited this study to the most commonly reported patient outcome measures, namely, the Western Ontario McMaster Universities Osteoarthritis Index (WOMAC) total score and the Visual Analogue Scale (VAS) for pain.

The purpose of this study was to perform a comprehensive, systematic review and meta‐analysis of the outcomes of PRP compared to HA based on patient reported outcome scores (PROs) of the WOMAC combined score and the VAS, limiting the analysis only to 6 months and 12 months of follow‐up and including only randomised controlled trials. Our hypothesis was that PRP would have a greater benefit than HA for the treatment of knee osteoarthritis based on common patient reported outcome measures. We used these time periods since 6 months is a reasonable expected period of relief for patients who are willing to undergo multiple injections and blood draws that are often not covered by health insurance. The one‐year time point was selected, as it is a best‐case scenario of effect for an intra‐articular injection and very few studies consistently report data for longer than 1 year. We further attempted to distinguish this study from other similar reviews by classifying the PRP used in each study based on the platelets, activation, white cell classification (PAW) [9]. This classification highlights the high variability in the studies on this clinically important topic and provides a realistic framework for the reader.

## METHODS

### Protocol and registration

The protocol was registered prospectively with PROSPERO, CRD42021218023.

#### Eligibility criteria

The systematic review was conducted according to the Preferred Reporting Items for a Systematic Review and Meta‐Analyses (PRISMA) guidelines and checklist. Studies were eligible for inclusion if they were a prospective randomised clinical trial comparing PRP and HA and they included patient reported outcomes (PROs). Studies that also included other treatments such as saline or corticosteroids were included but only the PRP and HA groups were included in the analyses. Patient reported outcomes that were assessed in this study included the WOMAC combined score and the VAS at baseline, at 6 months, and at 12 months. Exclusion criteria included non‐systematic, systematic reviews, network meta‐analyses, randomised controlled trials (RCTs) of PRP with other control groups, procedures combined with surgical treatments, combination treatments, a critical analysis of the raw data of a previously published and controversial RCT (which we also excluded from our analysis), animal studies, and basic science studies. RCTs that met the initial criteria were excluded secondarily if their PROs were not presented in a usable format or were modified, if they had only short‐term follow‐up (e.g., 3 months), if they were an update on an already included RCT, if they had injections in bilateral knees, or if they had a small number of subjects per group (10 or less per group).

Two independent reviewers agreed on the search criteria (AAJ and DD). A search was then performed on the following databases: Embase, PubMed, Scopus and Cochrane databases from inception to 04/29/2023.

The Embase, Scopus, and PubMed databases were searched with two searches with slight variation in search terms, Search #1 and Search #2. Search #1 was for the terms ‘Platelet Rich Plasma’ and ‘osteoarthritis’ and ‘knee’. Search #2 was for the terms ‘PRP’ and ‘osteoarthritis’ and ‘knee’. The Cochrane database was organised slightly differently and as a result the search was for ‘platelet‐rich plasma’ and ‘knee osteoarthritis’. The series of studies was searched within the reference management software, Endnote X4 (Thompson‐Reuters, Toronto, CA) for the keyword terms ‘randomised’, ‘RCT’, or ‘randomised controlled trial’.

**Figure 1:**
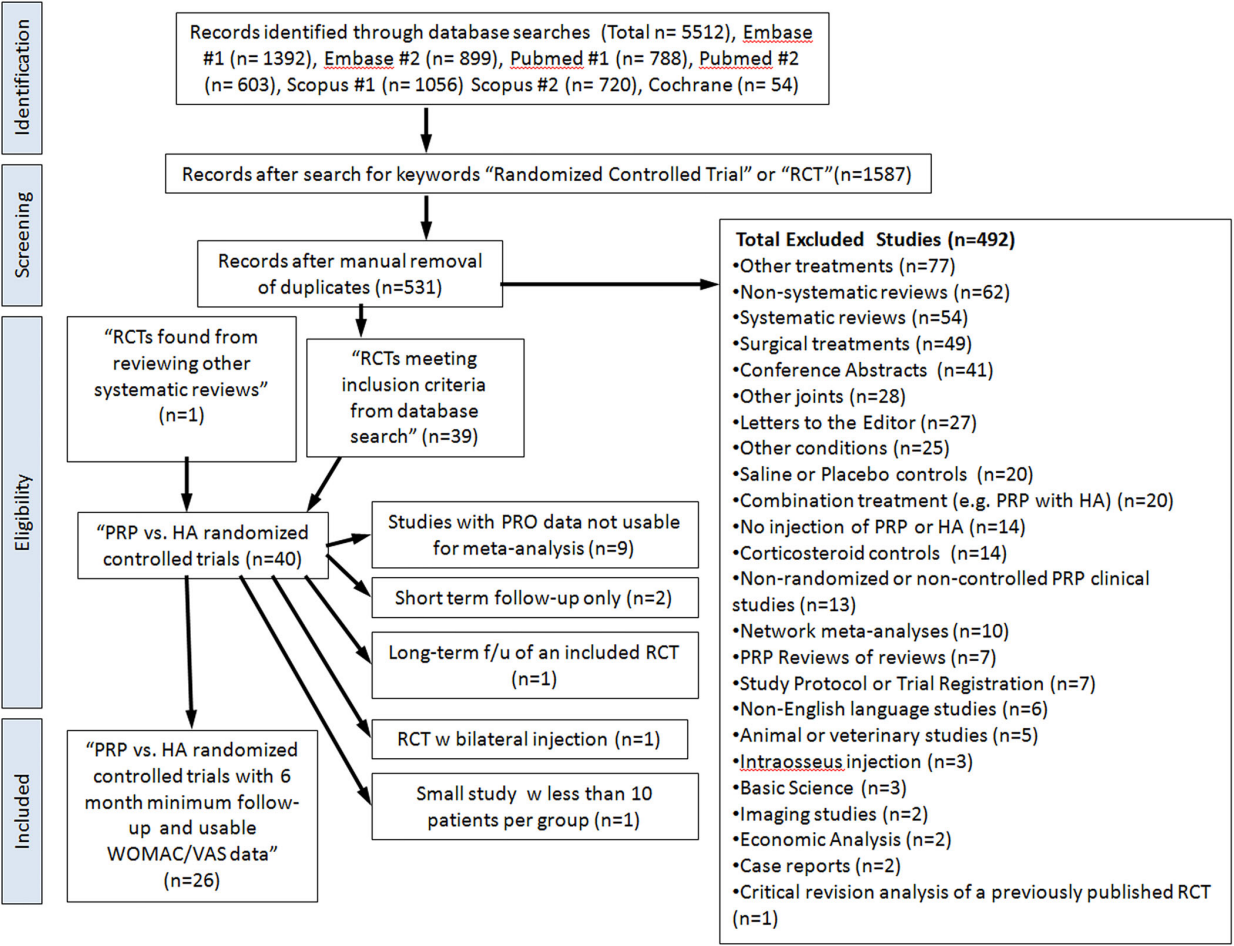
Study selection process.

The eligibility of studies for inclusion in this review was assessed by two independent reviewers with a long history of clinical experience with both PRP and HA injections for knee osteoarthritis. Exclusion criteria and number of excluded studies are presented in Figure 1. This was performed in an unblinded fashion. Disagreements were resolved by consensus meeting of the two senior clinician authors.

Data was extracted directly from the studies and placed in a spreadsheet document managed by the two senior clinician authors (Excel, Microsoft, Redmond, WA). Demographic and study characteristics including gender breakdown, follow‐up range are presented in Table 1. A summary of patient reported outcomes and adverse events is shown in Table 2. Details of the HA and PRP preparations, injection protocol, and PRP classification are provided in Table 3.

**Table 1:** Study demographics and follow‐up.

	Author	Year	Single/double blinding	Total patients	Males	Females	PRP Group total patients	PRP Group #1 M/F	HA Group total patients	HA Group 1 M/F	Follow‐up range
1	Ahmad	2018	Single	89	28	61	45	14/31	44	14/30	3, 6 months
2	Arliani	2021	Non‐blinded	29	5	24	14	3/11	15	2/13	1, 3, and 6 months
3	Bansal	2021	Double	132	81	51	64	39/25	68	42/26	1, 3, 6 and 12 months
4	Basnaev	2021	Non‐blinded	128	Not specified	Not specified	47	Not specified	38	Not specified	1, 3 and 6 months
5	Buendia‐Lopez	2018	Single	65	31	34	33	16/17	32	15/17	6, 12 months
6	Cerza	2012	Non‐blinded	120	53	67	60	25/35	60	28/32	1, 3, 6 months
7	Cole	2017	Double	97	48	51	49	28/21	50	20/30	1.5, 3,6,12 months
8	Dulic	2021	Double	64	28	36	34	15/19	30	13/17	1, 3, 6, 9, and 12 months
9	Duymus	2017	Non‐blinded	67	2	65	33	1/32	34	1/33	1, 3, 6, 12 months
10	Filardo	2015	Double	183	112	71	94	60/34	89	52/37	2, 6, 12 months
11	Gormeli PRP 1	2017	Double	83	72	90	44	19/25	39	17/22	1.5 months, 3 months, and 6 months
11	GormeliPRP 3	2017	Double	78	72	90	39	16/23	39	17/22	1.5 months, 3 months, and 6 months
12	Huang, Y.	2019	Non‐blinded	80	44	36	40	25/15	40	19/21	3, 6, 9, 12 months
13	Kucukakkas	2022	Single	40	10	30	20	4/16	20	6/14	1 and 6 months
14	Lin	2019	Double	60	19	41	31	9/22	29	10/19	2, 6, 12 months
15	Louis	2018	Double	48	25	23	24	14/10	24	11/13	1, 3, 6 months
16	Park	2021	Double	110	24	86	55	16/39	55	8/47	6 weeks, 3 months, 6 months
17	Raeissadat	2015	Non‐blinded	139	23	116	77	8/69	62	15/47	2, 6, 12 months
18	Raeissadat	2017	Single	69	13	56	36	7/29	33	6/27	2, 6 months
19	Raeissadat	2020	Single	102	29	73	50	14/36	52	15/37	2 months, 6 months, and 12 months
20	Shoma	2021	Single	133	56	77	65	27/38	68	29/39	3 and 6 months
21	Spakova	2012	Non‐blinded	120	64	56	60	33/27	60	31/29	3, 6 months
22	Su	2018	Non‐blinded	55	23	32	25	11/14	30	12/18	1, 3, 6, 12, 18 months
23	Swedowski	2022	Non‐blinded	49	10	40	25	5/20	24	4/20 or 5/19	6, 12, and 26 weeks
24	Vaquerizo	2013	Double	96	38	58	48	16/32	48	22/26	6, 12 months
25	Wang	2022	Single	85	20	65	42	11/31	43	9/34	3, 6, and mean of 78.9 months
26	Yaradilmis (LEUK. POOR PRP	2020	Double	60	7	53	30	3/27	30	4/26	2, 6, 12 months
26	Yaradilmis (LEUK. RICH PRP	2020	Double	60	8	52	30	4/26	30	4/26	2, 6, 12 months

Abbreviations: HA, hyaluronic acid; PRP, platelet rich plasma.

**Table 2:** Patient reported outcomes.

	Author	Year	WOMAC PRP group (baseline)	WOMAC HA group (baseline)	WOMAC PRP group (6 months)	WOMAC HA group (6 months)	WOMAC PRP group (12 months)	WOMAC HA group (12 months)	VAS PRP group (baseline)	VAS HA group (baseline)	VAS PRP group (6 months)	VAS HA group (6 months)	VAS PRP group (12 months)	VAS HA group (12 months)	Adverse events	Dropouts by group
1	Ahmad	2018	N/A	N/A	N/A	N/A	N/A	N/A	58 ± 19	61 ± 17	41.4 ± 14.4	59.5 ± 15.2	N/A	N/A	N/A	1 Death in control group, 0 (PRP‐IAI), 1 (HA‐IAI)
2	Arliani	2021	42.5 ± 17.9	41.1 ± 15.5	41.1 ± 24.8	35.7 ± 35.7	N/A	N/A	N/A	N/A	N/A	N/A	N/A	N/A	None observed	None
3	Bansal	2021	54.97 ± 9	53.56 ± 7	36.7 ± 6.9	54.7 ± 8.4	51.94 ± 7.35	57.33 ± 8.92	N/A	N/A	N/A	N/A	N/A	N/A	Both groups had equal numbers of patients with mild transient adverse events.	11 PRP, 7 HA lost to followup
4	Basnaev	2021	N/A	N/A	N/A	N/A	N/A	N/A	68 ± 1	67 ± 3	43 ± 2	48 ± 1	N/A	N/A	None reported	None reported
5	Buendia‐Lopez	2018	42.57 ± 7.3	42.62 ± 7.3	33.6 ± 1.2	37.34 ± 1.2	34.51 ± 1.2	42.65 ± 0.9	61.5 ± 11	60.6 ± 9	49.0 ± 5.2	52.1 ± 6	50.3 ± 17	62.5 ± 4	2 Adverse events in HA group, led to their withdrawing from the study	2 dropouts in PRP group, 4 dropouts in HA group, 2 dropouts in NSAID group
6	Cerza	2012	76.9 ± 9.5	75.4 ± 10.7	36.5 ± 17.9	65.1 ± 10.6	N/A	N/A	N/A	N/A	N/A	N/A	N/A	N/A	None observed	No dropouts
7	Cole	2017	N/A	N/A	N/A	N/A	N/A	N/A	57.2 ± 21	62.9 ± 21	34.6 ± 23	48.6 ± 26	44 ± 32	57.3 ± 27	N/A	3 in PRP, 9 in HA
8	Dulic	2021	48.12 ± 17.02	46.41 ± 14.98	31.21 ± 21.26	37.71 ± 16.31	31.06 ± 23.34	35.29 ± 17.39	N/A	N/A	N/A	N/A	N/A	N/A	None observed	2 PRP, 3 HA
9	Duymus	2017	76.1 ± 9.4	77.0 ± 2.5	42.8 ± 7.1	44.5 ± 6.6	54.9 ± 10.8	69.3 ± 4.3	74 ± 10	83 ± 4	40 ± 13	43 ± 13	51 ± 13	68 ± 1	N/A	6 in PRP, 5 in HA,
10	Filardo	2015	N/A	N/A	N/A	N/A	N/A	N/A	26.8 + /‐ 12.0	28.4 ± 13.4	23.8 ± 12.9	25.9 ± 15.1	22.4 ± 11.1	26.6 ± 15.2	2 patients in HA group reported severe pain and swelling	2 in PRP, 4 in HA, 4 (PRP group), 11 (HA group)
11	Gormeli PRP 1	2017	N/A	N/A	N/A	N/A	N/A	N/A	49.7 ± 5.8	49.5 ± 4.6	38 ± 6.3	39.2 ± 7.2	N/A	N/A	Mentioned, but not specified	PRP3: 5 dropouts, 2 withdrew consent; PRP1: 1 dropout; HA: 5 dropouts, 2 withdrew; Saline: 3 dropouts, 2 withdrew
11	GormeliPRP 3	2017	N/A	N/A	N/A	N/A	N/A	N/A	49.7 ± 5.2	49.5 ± 4.6	28.6 ± 10.8	39.2 ± 7.2	N/A	N/A	Mentioned, but not specified	PRP3: 5 dropouts, 2 withdrew consent; PRP1: 1 dropout; HA: 5 dropouts, 2 withdrew; Saline: 3 dropouts, 2 withdrew
12	Huang, Y.	2019	48.19 ± 4.96	47.23 ± 5.37	21.14 ± 5.17	26.38 ± 5.20	16.10 ± 7.22	30.64 ± 8.36	45.7 ± 6.10	45.4 ± 5.96	N/A	N/A	19.8 ± 14.37	21.4 ± 15.23	2 HA, and 5 PRP patients reported pain	No dropouts
13	Kucukakkas	2022	50.8 ± 20.4	53.4 ± 13.1	35.0 ± 27.4	41.1 ± 14.7	N/A	N/A	67.5 ± 21	74 ± 15	47.5 ± 29	49 ± 21	N/A	N/A	None observed	3 PRP, 2 HA
14	Lin	2019	45.30 ± 17.14	45.44 ± 17.34	36.21 ± 17.73	45.22 ± 18.97	34.84 ± 19.84	48.64 ± 20.65	N/A	N/A	N/A	N/A	N/A	N/A	No major complications; only transient local pain	1 Knee in PRP; 2 knees in HA;
15	Louis	2018	36.5 ± 16.8	34.7 ± 21.8	27.4 ± 21.5	26.6 ± 24.2	N/A	N/A	48 ± 23	51 ± 22	40 ± 25	35 ± 28	N/A	N/A	Knee sprain and amygdalotomy in HA group, & knee sprain in PRP group. group.	4 (PRP group), 8 (HA group) lost to followup
16	Park	2021	38.6 ± 15.4	33.0 ± 11.8	31.6 ± 18.2	26.8 ± 12.7	N/A	N/A	59.0 ± 9.9	55.2 ± 9.5	37.9 ± 21.6	41.2 ± 19.1	N/A	N/A	None observed	None
17	Raeissadat	2015	39.5 + /‐ 17.06	28.69 + /‐ 16.69	N/A	N/A	18.44 ± 14.35	27.46 ± 16.36	N/A	N/A	N/A	N/A	N/A	N/A	None observed	21 dropouts, 10 (PRP), 11 (HA)
18	Raeissadat	2017	42.9 ± 13.51	38.8 ± 12.62	24.4 ± 16.54	27.4 ± 11.38	N/A	N/A	78 ± 17.8	74 ± 14.8	46 ± 27.8	48 ± 23.9	N/A	N/A	Not mentioned	5 (PRGF), 3 (HA)
19	Raeissadat	2020	41.96 ± 11.71	39.71 ± 10.4	24.86 + 14	28.35 ± 9.4	27.10 ± 12.3	32.41 ± 11.8	78 ± 15	78 ± 11	43 ± 23	47 ± 19	45 ± 17	61 ± 18	Minor complications related to injection, 19.6% PRGF, 5.9% HA.	10 PRGF, 7 HA
20	Shoma	2021	N/A	N/A	N/A	N/A	N/A	N/A	76 ± 6	74 ± 5	33 ± 4	36 ± 5	N/A	N/A	None observed	10 PRP, 7 HA
21	Spakova	2012	38.76 ± 16.5	43.21 ± 13.7	18.85 ± 14.09	30.90 ± 16.57	N/A	N/A	N/A	N/A	N/A	N/A	N/A	N/A	Six cases: temporary mild worsening of pain in injection of PRP for <2 days	None
22	Su	2018	50.17 ± 1.6	49.88 ± 1.54	34.37 ± 1.22	38.84 ± 1.60	39.97 ± 2.93	43.40 ± 2.35	70.2 ± 2.7	70.4 ± 3.3	42.6 ± 3.5	44.4 ± 6.4	47.8 ± 1.9	54.5 ± 3.8	None observed	None
23	Swedowski	2022	53.84 ± 14.96	53.92 ± 15.19	23.4 ± 6.35	34.87 ± 4.20	N/A	N/A	N/A	N/A	N/A	N/A	N/A	N/A	None observed	0 PRP, 1 HA
24	Vaquerizo	2013	45.9 ± 12.7	50.8 ± 18.4	27.2 ± 15.1	50.4 ± 23.2	30.8 ± 15.5	54.2 ± 19.2	N/A	N/A	N/A	N/A	N/A	N/A	16 total adverse events, mostly pain at infiltration site	4 withdrew.
25	Wang	2022	37.8 ± 13.7	37.5 ± 11.4	22.5 ± 8	31.8 ± 12.1	N/A	N/A	53 ± 18	51 ± 15	41.7 ± 14.3	48.4 ± 18.5	N/A	N/A	N/A	None at 6 months
26	Yaradilmis (LEUK. POOR PRP	2020	81.57 ± 13.74	79.17 ± 13.27	43.33 ± 15.55	44.17 ± 12.01	49.83 ± 19.83	51.33 ± 21.89	88.3 ± 12.1	87.7 ± 12.2	29.7 ± 14.8	37.0 ± 25.1	41.7 ± 23.4	49.7 ± 26.7	12 pts in group LR‐PRP, 3 pts in group LP‐PRP, 2 pts in HA group w/local reactions	6 total dropouts
26	Yaradilmis (LEUK. RICH PRP	2020	82.23 ± 8.37	79.17 ± 13.27	32.00 ± 17.00	44.17 ± 12.01	35.83 ± 19.35	51.33 ± 21.89	89.3 ± 9.4	87.7 ± 12.2	18.3 ± 20	37.0 ± 25.1	22.3 ± 23.3	49.7 ± 26.7	12 pts in group LR‐PRP, 3 pts in group LP‐PRP, 2 pts in HA group w/local reactions	6 Total dropouts

Abbreviations: HA, hyaluronic acid; NSAID, non‐steroidal anti‐inflammatory drugs; PRGF, plasma rich in growth factors; PRP, platelet rich plasma; VAS, Visual Analogue Scale; WOMAC, Western Ontario McMaster Universities Osteoarthritis Index.

**Table 3:** Injection characteristics of HA and PRP interventions including PAW classification [9].

	Author	Year	HA	PRP group	PRP type (PRP, ACP, PRGF, etc.)	Blood volume needed	Preparation time	Single/double spin	PRP frozen (Y/N)	System Utilised	Total # Injections	Interval between PRP injections	Platelet Concentration	Anticoagulant needed	Exogenous Activation	White blood cells	PAW Classification
1	Ahmad	2018	20 mg of HA, (brand not specified), once every other week for 3 weeks	3 injections of PRP	PRP	8 mL	>9 min	Single	N	Self‐made	3	2 Weeks	n/a	n/a	n/a	Present	?‐?‐?
2	Arliani	2021	Synvisc One, 1 injection	1 injection of PRP	PRP	15 mL	5 minutes	Single	N	Arthrex ACP	1	n/a	Not specified	n/a	n/a	n/a	P2‐Bβ
3	Bansal	2021	4 ml of Monovisc (Anika, MA, USA)	1 injection of PRP	PRP	60 mL	10 mins (first spin), 15 mins (second spin)	Double	N	Self‐made	1	n/a	2.3× 10^5 platelet/µl	ACDA	n/a	Leucocyte Poor	P2‐Bβ
4	Basnaev	2021	Hyaluronic Acid (not specified) once a week for 4 weeks.	4 injections of PRP	PRP	not specificed	Not specified	Not specified	N	Self‐made	4	1 week	800 ×10^9 per liter	n/a	n/a	n/a	P3‐?‐?
5	Buendia‐Lopez	2018	Durolane©, single injection (Bioventus, The Netherlands)	1 injection of PRP	PRP	60 mL	>25 min	Double	N	Self‐made	1	n/a	1.095 ×10^6 + /− 23,200 (3.87X normal)	n/a	CaCl_2_	Leucocyte Poor	P3‐x‐Bβ
6	Cerza	2012	Hyalgan (4 weekly injections)	4 injections of PRP	ACP	12 mL	Not specified	Not specified	Not in article	Arthrex ACP	4	Weekly	Not specified	ACDA	n/a	Leucocyte Poor	P2‐Bβ
7	Cole	2017	Synvisc, 3 injections once a week	3 injections of PRP	ACP	10 mL	>5 min	Single	N	Arthrex ACP	3	Weekly	1.73X in PRP vs peripheral blood	n/a	n/a	Leucocyte Poor	P2‐Bβ
8	Dulic	2021	Cartinorm® 2 ml (Goodwill Pharma, Hungary (3 injections)	1 injection of PRP	PRP	60 mL	Not specified	Double	N	Arthrex Angel	1	n/a	2179.31 × 10^6 (7.23X baseline)	ACDA	n/a	Leucocyte rich	P4‐Aα
9	Duymus	2017	Ostenil Plus (one injection)	2 injections of PRP	PRP	14 mL	>7 min	Single	N	Y‐cellbio	2 (PRP group), 1 (HA group)	1 Month	Not specified	Unspecified	n/a	n/a	P4 ‐?
10	Filardo	2015	Hyalubrix (Fidia), 3 injections Fidia	3 injections of PRP	ACP	150 mL taken, actually need 110 mL	>21 min	Double	Y	Self‐made	3	Weekly	mean of 4.6 + /− 1.4 times that of baseline blood values	n/a	CaCl_2_	1.1 + /− 0.5 X whole blood	P3‐x‐Aα
11	Gormeli	2017	Orthovisc, once weekly for 3 weeks.	1 injection of PRP	PRP	150 mL taken, actually need 110 mL	>18 min	Double	N	Self‐made as described in Filardo (2012)	1 (PRP), 3 (HA)	Weekly	5.2 o5 5.3X Baseline	ACDA	CaCl_2_	n/a	P4‐x‐Aα
11	Gormeli	2017	Orthovisc, once weekly for 3 weeks.	3 injections of PRP	PRP	150 mL taken, actually need 110 mL	>18 min	Double	First injected immediately, other two frozen	Self‐made as described in Filardo (2012)	3 (PRP), 3 (HA)	Weekly	5.2 o5 5.3X Baseline	ACDA	CaCl_2_	n/a	P4‐x‐Aα
12	Huang, Y.	2019	HA, SK chemical research co., LTD, Tokyo, Japan, once weekly for 3 weeks	1 injection of PRP	?	8 mL	>5 min	Single	N	Not specified	1 (PRP), 3 (HA)	n/a	“two‐fold increase”, At least 1.0 × 10^6 platelets per μl	n/a	n/a	Below normal levels	P2‐Bβ
13	Kucukakkas	2022	Reviscon Mono, 48 mg (VSY Biotechnology, Amsterdam, Netherlands), 1 injection	1 injection of PRP	PRP	150 mL	10 mins (first spin), 15 mins (second spin)	Double	N	Sakura Medikal, Istanbul, Turkey	1	n/a	7.66 ± 3.49 X baseline	n/a	n/a	Equal to whole blood	P4‐Bβ
14	Lin	2019	Hyruan Plus, LG Chem, Seoul, Republic of Korea, once weekly for 3 weeks	3 injections of PRP	PRP	10 mL	8 minutes	Single	N	RegenKit‐THT; Regen Lab	3	Weekly	1.81 + /− 0.34X normal	ACDA	n/a	Leucocyte Poor	P2‐Bβ
15	Louis	2018	Durolane, one injection	1 injection of PRP	PRP	52.2 mL for men; 37.5 mL for women	>30 min	Double	N	Self‐made	1	n/a	3.3 ± 0.7 X Baseline	n/a	n/a	Leucocyte Poor	P2‐Bβ
16	Park	2021	Synovian; 30 mg/3 mL,(LG Life Sciences. Seoul, Republic of Korea)	1 injection of PRP	PRP	54 mL	15 minutes	Single	Y	(GPS III; Zimmer Biomet	1	n/a	3X normal	ACDA	n/a	Leucocyte Rich	P3‐Aα
17	Raeissadat	2015	3 Injections of Hyalgan (Fidia)	2 injections of PRP	PRP	35 – 40 mL	>22 min	Double	N	Rooyagen (Arya Mabna Tashkhis Corporation, Iran)	2 (PRP), 3 (HA)	4 Weeks	5X blood platelet concentration	ACDA	n/a	Leucocyte Rich	P4‐x‐Aα
18	Raeissadat	2017	3 Injections of Hyalgan (Fidia)	2 injections of PRP	PRGF	35 mL	15 minutes (first spin), 7 minutes (second spin), then 3rd centrifuge to remove platelets	Triple	N	Rooyagen (Arya Mabna Tashkhis Corporation, Iran)	2 (PRGF), 3 (HA)	3 Weeks	No Platelets	ACDA	Epinephrine and CaCl_2_	No WBC	P2‐x‐Bβ
19	Raeissadat	2020	3 Injections of Hyalgan (Fidia)	2 injections of PRP	PRGF	35 mL	15 minutes (first spin), 7 minutes (second spin), then 3rd centrifuge to remove platelets	Triple	N	Rooyagen (Arya Mabna Tashkhis Corporation, Iran)	2 (PRGF), 3 (HA)	3 Weeks	No Platelets	ACDA	Epinephrine and CaCl_2_	No WBC	P1‐x‐Bβ
20	Shoma	2021	Single dose intra‐articular HA (Sylocet) (4 ml)	1 injection of PRP	PRP	not specificed	Not specified	Not specified	N	Self‐made	1	n/a	n/a	n/a	n/a	n/a	?‐?‐?
21	Spakova	2012	3 Injections of HA (Erectus 1.2%; CSC Pharmaceuticals Handels GmbH),	3 injections of PRP	PRP	27 mL	>25 min, 15 mins (first spin), 10 mins (second spin)	Triple	N	Self‐made	3 (PRP), 3 (HA)	Weekly	4.5X normal blood	ACDA	n/a	Leucocyte Rich, 3.6X normal	P2‐Aα
22	Su	2018	2 ml of HA (Freda,Shandong, China), 5 weekly doses Shandong, China),	2 injections of PRP	PRP	45 mL	>21 min	Double	N	Self‐made	PRP 2 injections, HA 5 injections	2 Weeks for PRP	5.6X normal	ACDA	CaCl_2_	Leucocyte Rich	P3‐x‐Aα
23	Swedowski	2022	Biovisc Ortho Single, 30 mg/mL, molecular weight 3.400–3.800 kDa, Atradis, Poland	1 injection of PRP	PRP	10–12 mL	5 minutes	Single	N	Density Platelet Gel, IBF, Scafati, Italy	1	n/a	n/a	n/a	n/a	n/a	?‐?‐?
24	Vaquerizo	2013	Durolane©, single injection (Bioventus, The Netherlands)	1 injection of PRP	PRGF	363 mL	8	Single	N	Self‐made	1 (PRP), 1 (HA)	Once	No Platelets	ACDA	CaCl_2_	No WBC	P2‐x‐Bβ
25	Wang	2022	ARTZ, Sodium Hyaluronate injection; 25 mg/2.5 ml; Seikagaku Corporation	3 injections of PRP	PRP	40 mL	10 mins (first spin), 10 mins (second spin)	Double	N	Self‐made	3	1 week	6.1X normal	Unspecified	CaCl_2_	Leucocyte Rich	P3‐x‐Aα
26	Yaradilmis	2020	Ostenil®, once a week for 3 weeks	3 injections of Leucocyte‐poor PRP	PRP	25 mL	>15 min, >25 min, 5 mins (first spin), 10 mins (second spin)	Single	N	Self‐made	3 per group	Weekly	1.9X	ACDA	n/a	Leucocyte Poor	P2‐Bβ
26	Yaradilmis	2020	Ostenil®, once a week for 3 weeks	3 injections of Leucocyte‐rich PRP	PRP	25 mL	>15 min, >25 min, 5 mins (first spin), 10 mins (second spin)	Double	N	Self‐made	3 per group	Weekly	4.6X	ACDA	n/a	Leucocyte Rich	P3‐Aα

Abbreviations: HA, hyaluronic acid; PAW, platelets, activation, white cell classification; PRGF, plasma rich in growth factors; PRP, platelet rich plasma.

Risk of bias was assessed using the Cochrane Collaboration's RoB 2 tool for assessing risk of bias in randomised trials [39]. Risk of bias was assessed by pairs of reviewers including one clinician senior author in each pair. The summary measures included were validated patient reported outcomes that were available at baseline and at 6 months and 12 months minimum follow‐up. These PROs included the WOMAC combined score on a scale of 0‐96 and the VAS. The difference in means was used as the effect measure for meta‐analysis. The WOMAC was only included if the entire score was provided.

Meta‐analysis was performed using random‐effects models. Inverse variance weighted mean differences were used to estimate pooled effect sizes with 95% confidence intervals. Statistical heterogeneity was assessed by *χ*² tests using the Q statistic, with the I² statistic to quantify heterogeneity.

Forest plots were generated for the meta‐analysis. The meta‐analysis was performed at 6 months and 12 months follow‐up for the WOMAC combined and VAS scores. In order to determine the effect of platelet count on clinic scores, studies were grouped according to the platelet count of the PRP used based on the PAW classification. In this classification, P1 is less than baseline, P2 is greater than baseline up to 750,000 platelets/μL, P3 is between 750,000 and 1,250,000 platelets/μL, and P4 is greater than 1,250,000/μL [9]. In this systematic review, all studies that were classified as P1 were treatments with plasma rich in growth factors (PRGF) where platelets were activated but the actual platelet cells were removed.

Funnel plots were generated to assess for publication bias in assessing for asymmetry which could result from non‐publication of small trials or trials with negative results. The asymmetry of these plots was assessed through visual assessment.

## RESULTS

### Study selection

The total number of references for each database was as follows: Embase Search #1 (Platelet Rich Plasma), 1392 abstracts, Embase Search #2 (PRP), 899 abstracts, PubMed Search #1 (Platelet Rich Plasma), 788 abstracts, PubMed Search #2 (PRP), 603 abstracts, Scopus Search #1 (Platelet Rich Plasma), 1056 abstracts, Scopus Search #2 (PRP), 720 abstracts. The Cochrane Register of Controlled Trials database was searched on 4/23/2023 with the search terms ‘Platelet‐rich‐plasma’ and ‘Knee Osteoarthritis’ in keyword search revealing 54 abstracts. This led to a total of 5512 abstracts. The series of studies was searched within the reference management software, Endnote X4 (Thompson‐Reuters, Toronto, CA) for the keyword terms ‘randomised’, ‘RCT’, or ‘randomised controlled trial’. After further selection of studies in the Endnote software for those keywords the pool was narrowed to 1587 abstracts. After removal of duplicates in the software, we obtained 531 abstracts.

These were further categorised by the two senior clinician authors (AAJ and DD) for the inclusion criteria for this study were randomised prospective studies in patients with knee osteoarthritis treated with PRP and a control group of HA with a follow‐up of 6 months and 12 months. These criteria narrowed the group of studies to 39 studies. Other references were evaluated for any additional RCTs that would fit the inclusion criteria. One RCT was identified in this fashion [45]. After further analysis, fourteen of the studies were excluded due to unusable data such as scores without standard deviations (*n* = 9) [15, 26, 29, 32, 35, 37, 44, 47, 49], short‐term follow‐up only (*n* = 2) [24, 43], long term follow‐up of an already included RCT (*n* = 1) [10], bilateral knee injections (*n* = 1) [36] or a small study with 10 or less knees per group (*n* = 1) [22].

In the end, 26 studies were included in the final meta‐analysis. These included 28 distinct comparisons. Two studies had two independent comparisons to a control group [18, 48]. All studies were randomised controlled trials. They included a total of 1650 knees. A summary of the study selection is provided in Figure 1.

One paper included only a WOMAC pain score rather than the full score [8]. Its WOMAC data were excluded. In another study, a modified WOMAC score was used on a scale of 0–100 with a higher score being a better outcome [28]. To correct that to the standard WOMAC, their scores were subtracted from 100 and then multiplied by a correction of 0.96. In some studies, the data of interest were included in a graphical form rather than in the text. In these cases, the means and standard deviations were determined using image analysis software (Photoshop CS, Adobe, USA). For the VAS, all scores were standardised to a scale of 0–100 with 0 being no pain and 100 being maximal pain. Several studies used the EuroQol Visual Analogue Scale (EQ‐VAS scale) [14, 18]. The EQ‐VAS is a widely used general health instrument where 100 is the best imaginable health and 0 is the worst imaginable health. In these cases, the score was subtracted from 100 to standardise it with the other studies included in the VAS analysis [20, 23, 31]. Basnaev et al. provided two VAS's for analysis, one at ‘rest’ and one with ‘walking’ [3]. We included the more relevant VAS for ‘walking’ in the meta‐analysis. Kucukakkas provided two types of VAS scores as well, one at ‘rest’ and one with ‘movement’ [25]. We included the VAS with ‘movement’ in the meta‐analysis.

### Meta‐analysis results

**Figure 2:**
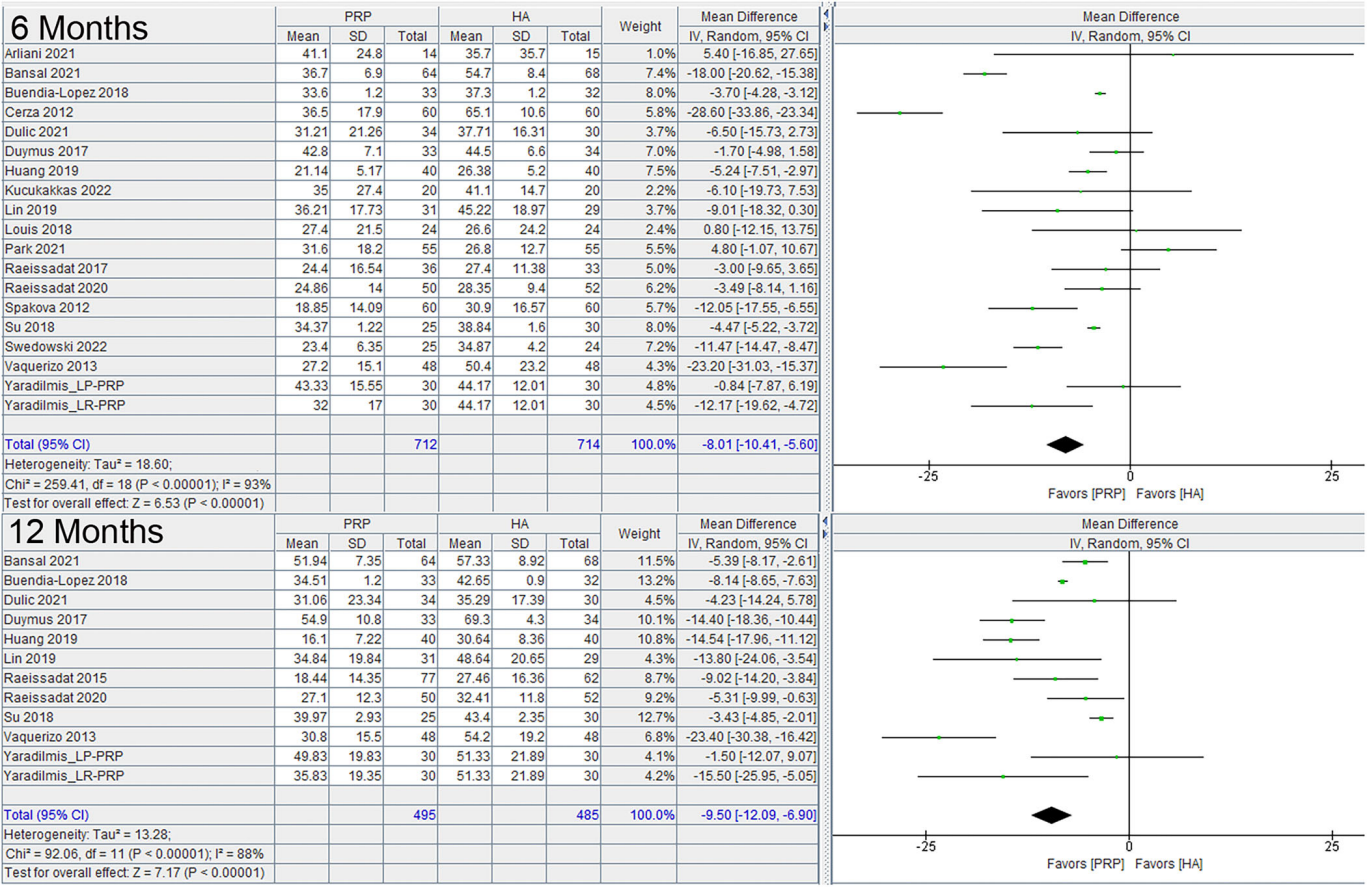
WOMAC summary score at 6 months, and 12 months comparing PRP and HA in RCTs. CI, confidence interval; HA, hyaluronic acid; PRP, platelet rich plasma; RCT, randomised controlled trial; WOMAC, Western Ontario McMaster Universities Osteoarthritis Index.

**Figure 3:**
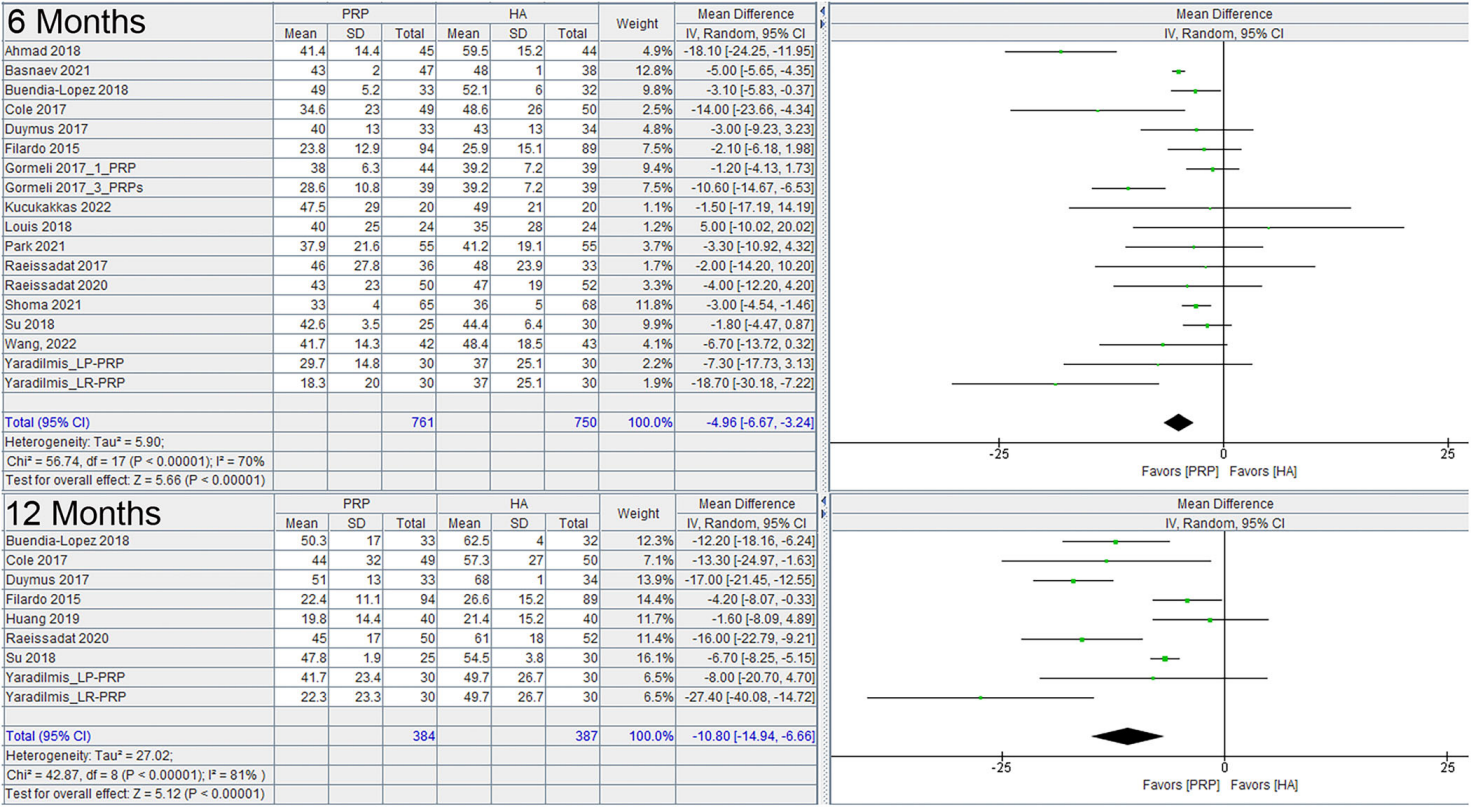
VAS at 6 months, and 12 months comparing PRP and HA in RCTs. CI, confidence interval; HA, hyaluronic acid; PRP, platelet rich plasma; RCT, randomised controlled trial; VAS, Visual Analogue Scale.

PRP had a significant benefit over HA based on the WOMAC at 6 months (−8.01, 95% C.I.: −10.41 to −5.60) and at 12 months (−9.50, 95% C.I.: −12.09 to −6.90) (Figure 2). It also had a significant benefit over HA on the VAS at 6 months (−4.96, 95% C.I.: −6.67 to −3.24) and at 12 months (−10.80, 95% C.I.: −14.94 to −6.66) (Figure 3). The analysis of baseline data for the WOMAC and for VAS scores did not significantly favour any one group.

### PRP characteristics and PAW classification of included studies

**Figure 4:**
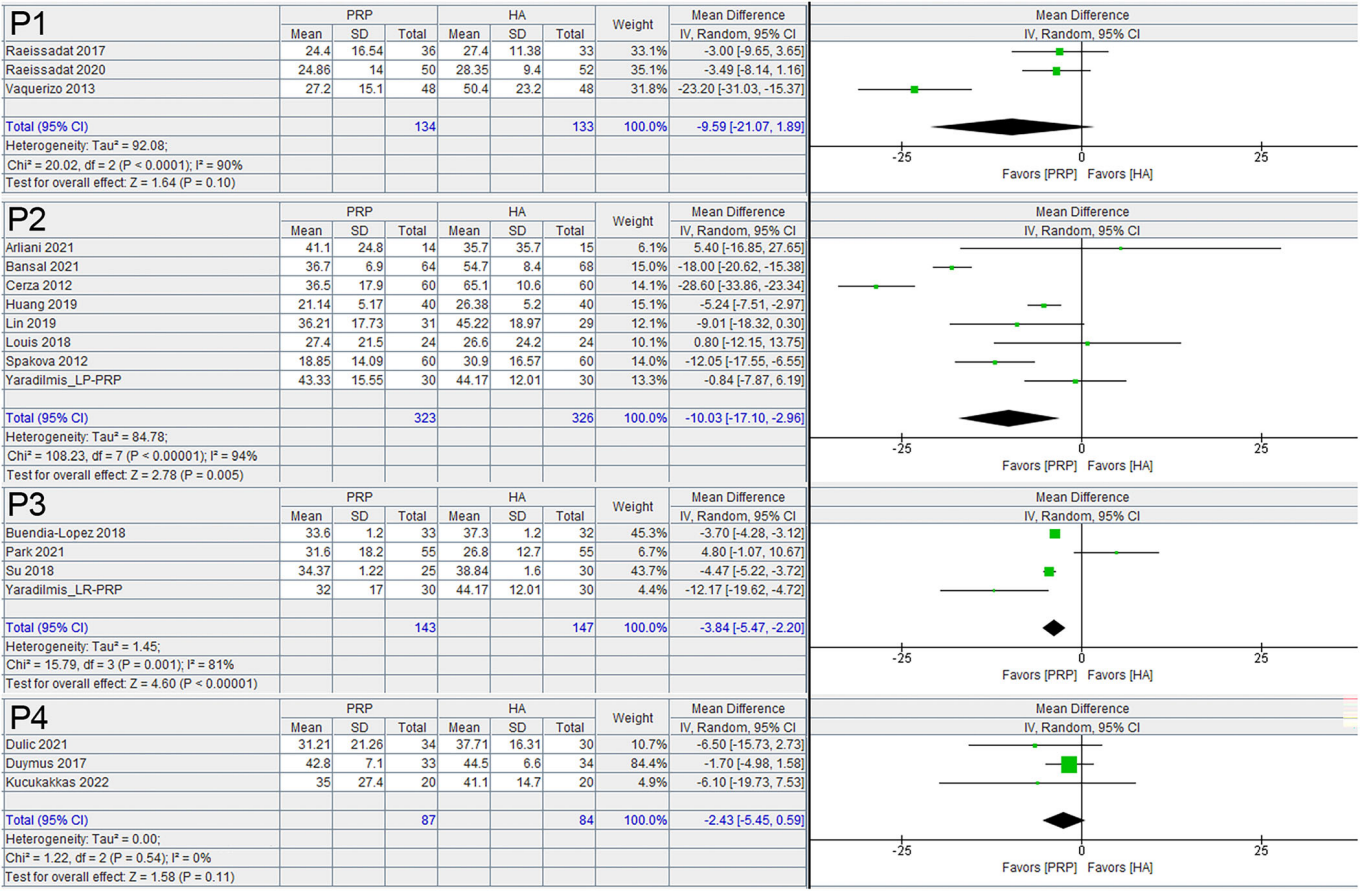
WOMAC summary scores at 6 months based on platelet concentration according to PAW Classification comparing PRP and HA in RCTs. CI, confidence interval; HA, hyaluronic acid; PRP, platelet rich plasma; RCT, randomised controlled trial; WOMAC, Western Ontario McMaster Universities Osteoarthritis Index.

**Figure 5:**
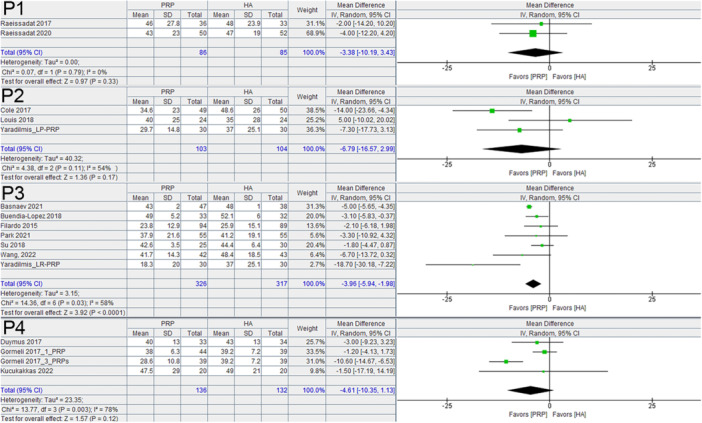
VAS scores at 6 months based on platelet concentration according to PAW Classification comparing PRP and HA in RCTs. CI, confidence interval; HA, hyaluronic acid; PRP, platelet rich plasma; RCT, randomised controlled trial; VAS, Visual Analogue Scale.

The included studies demonstrated a broad range of protocols for PRP preparation and injection. This led to variations in the PAW classification throughout the studies. Within the 28 comparisons (from the 26 studies), an anticoagulant was used in 17 of the comparisons, 15 of these were ACDA (anticoagulant citrate dextrose solution) and two were unspecified. In 11 of the comparisons, no anticoagulant was specified. An activator was used in 9 of the comparisons, all of which included calcium chloride, two of which also included epinephrine. In one comparison, there was no activator used, and in the remaining 18 comparisons, there was no specification of the presence or absence of an activator. Within the entire group of comparisons, the PAW classification was unknown in 3. Of the remaining 25 comparisons, there were 12 different separate classifications including P1‐x‐Bβ, P2‐Aα, P2‐Bβ, P2‐x‐Bβ, P3‐?‐?, P3‐Aα, P3‐x‐Aα, P3‐x‐Bβ, P4 ‐?, P4‐Aα, P4‐Bβ and P4‐x‐Aα. When stratified based on platelet concentration according to the definitions in the PAW classification and evaluated at the 6 month time interval, preparations with P2 and P3 platelet concentrations significantly favoured PRP over HA for the WOMAC score (Figure 4), with a mean difference of −10.03, 95% C.I.: −17.10 to −2.96 for P2 concentrations and a mean difference of −3.84, 95% C.I.: −5.47 to −2.20 for P3 concentrations. For the VAS, P3 platelet concentrations were the only subgroup that provided a statistically significant benefit over HA for the VAS score (Figure 5), with a mean difference of −3.96, 95% C.I.: −5.94 to −1.98).

#### Risk of bias

**Figure 6:**
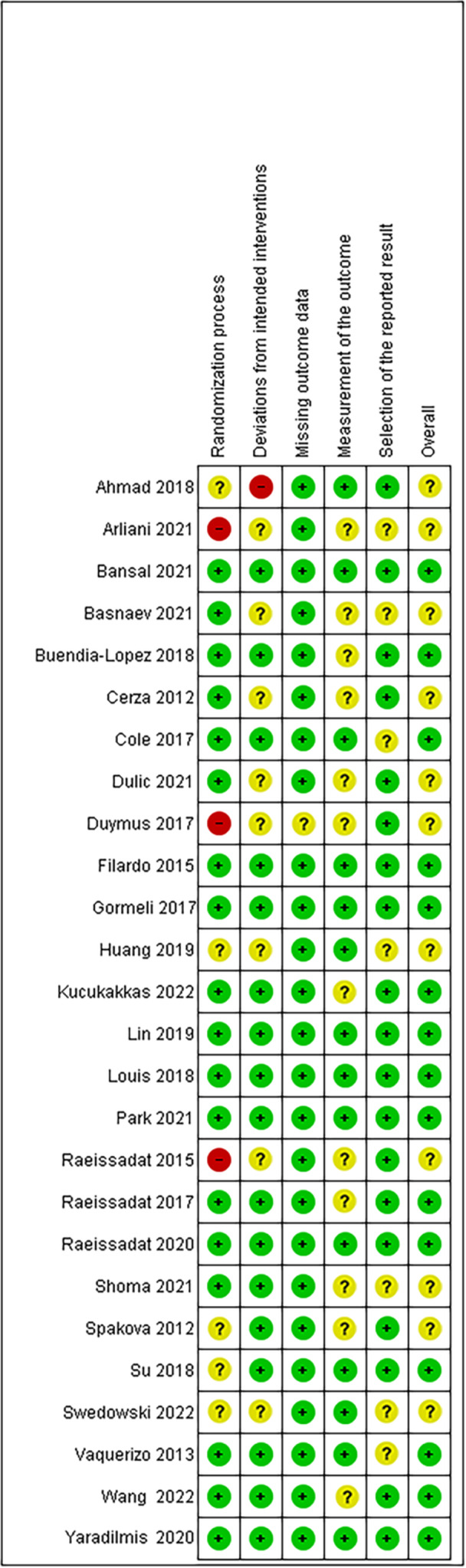
Risk of bias table according to the RoB 2 tool. (Green: 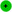, low risk of bias, Yellow: 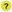, unknown risk, and Red: 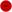, high risk of bias) [39].

**Figure 7:**
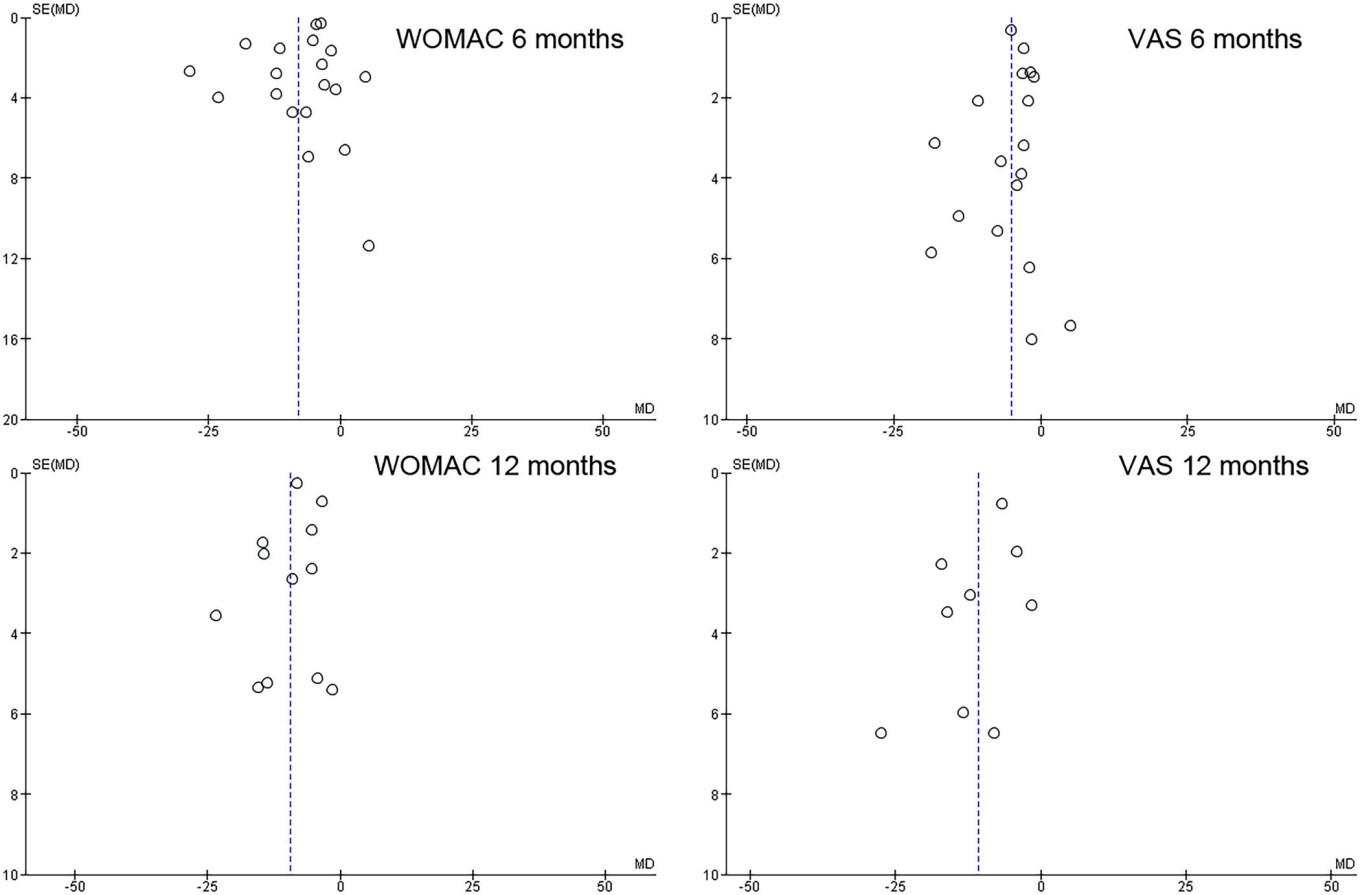
Funnel plots comparing WOMAC and VAS at 6 and 12 months demonstrating symmetric distribution of studies at 6 months, and 12 months indicative of low risk of publication bias. (SE of mean difference vs. mean difference). VAS, Visual Analogue Scale; WOMAC, Western Ontario McMaster Universities Osteoarthritis Index.

Overall risk of bias was low based on the methodology of the studies. The risk of bias across studies is provided in Figure 6. Funnel plots were generated demonstrating a symmetric distribution of points on either side of the mean difference line and shown in Figure 7. This indicates low risk of publication bias.

## DISCUSSION

In this systematic review and meta‐analysis, we have demonstrated that intraarticular injections of PRP for knee osteoarthritis have significantly better outcomes based on validated measures including the WOMAC and VAS scores at 6 and 12 months. Furthermore, we have attempted to specify the type of HA used and have classified all PRP's according to the PAW classification [9]. Using the PAW classification, we have found that the P2 and P3 platelet concentrations corresponding to ‘greater than baseline to 1,250,000 platelets/μL’ significantly favoured PRP over HA for the WOMAC score and platelet concentrations classified as P3, corresponding to ‘between 750,000 and 1,250,000 platelets/μL’ significantly favoured PRP over HA for the VAS score.

Studies that included other experimental groups were still included in this study as long as there was a direct comparison between at least one PRP group and one HA group. We found great variability in the type of preparation of PRP used, the injection approach, the number of injections, and the timing of administration. This likely contributes to the high heterogeneity noted in our analysis based on the *I* ^2^ statistic.

The literature contains a number of other systematic reviews and meta‐analyses on these interventions. Belk et al. published a meta‐analysis on this same topic [4]. Their study included 18 studies with 811 patients. Their search period ended before ours, in September 2019. As a result, fewer studies were included in their analysis. Their study subdivided the PRP into two groups, leucocyte‐rich and leucocyte‐poor. They provided data on other factors such as the approach to the injection and the use of ultrasound. Belk et al. have performed a more up to date meta‐analysis to include comparisons of PRP to HA and to bone marrow aspirate concentrate (BMAC) [5]. Their study included one RCT by Sdeek et al. in the meta‐analysis which did not contain any standard deviations in the main manuscript or in any supplementary materials and was therefore excluded from our study [37]. It also did not include another study which was an RCT that would have met their inclusion criteria [6]. In both of their studies, Belk et al. did not specify the exact type of HA used in each of their included RCTs or classify the PRP used in each study as we have performed in this report. There are many variables in each PRP preparation and simple categorisation based on the leucocyte content is not as descriptive as the full PRP classification according to the PAW classification.

Filardo et al. performed a systematic review and meta‐analysis of PRP compared to a number of controls including HA, ozone, saline, and corticosteroids [16]. Their study had a relatively comprehensive set of included RCTs but did not specify the exact RCTs included in each meta‐analysis. Critically, there was no information provided on the PRPs used in the included RCTs based on any classification system.

Trams et al. performed a broad systematic review and meta‐analysis of osteoarthritis treatment with PRP compared to various controls. The greatest weakness of that study was the grouping of short follow‐up outcomes such as Spakova et al. [38] at 3 months in the same meta‐analysis as very long term follow‐up papers such as Dulic et al. [12] at 12 months and Su et al. [40] at 18 months. This lack of consistency makes interpretation of their meta‐analysis problematic. Multiple other meta‐analyses have been performed in addition to the ones above [7, 21, 41, 42]. None of them provides discreet outcomes at specific time points of 6 months and 12 months, provide classification of the PRP used, or limit the included PROs to non‐modified scores.

This study is limited, as are all systematic reviews and meta‐analyses, by the quality of the included studies. We found that different studies have wide variations in the baseline scores for patients with knee osteoarthritis even with the same Kellgren and Lawrence classification inclusion criteria. This may indicate cultural variations among different patient cohorts as well as differences in pain tolerance, inflammatory factors, and body weight among the populations studied. Many of the authors of the included studies have financial conflicts related to their work as consultants or developers of specific PRP preparation systems or protocols. The financial incentives are a potential explanation for the multitude of different PRP recovery systems, tubes or centrifuge systems employed in the literature. This issue is further magnified when one considers the technical variations used by different clinics such as those involving solitary versus multiple injections, fresh versus frozen injections, and the wide spectrum of platelet and leucocyte concentrations in the PRP's used. These confounding factors are not limited to the PRP groups but also affect the HA groups in these studies. As shown in Table 3, there are variations in HA manufacturers based on molecular weight, country of manufacture, volume injected, number of injections, and duration between injections. There are likely other variables not included in the majority of the papers including the level of experience of the clinician, the injection approach, the use of ultrasound, and the post‐injection protocol.

In summary, this meta‐analysis has demonstrated that PROs of the WOMAC score and VAS are more favourable for PRP compared to HA for knee osteoarthritis based on the available RCTs. However, the methodology of the included studies is variable and valid conclusions through the use of meta‐analysis may be limited as a result. Our recommendation is for more standardisation of PRP preparation and injection protocols perhaps via a consensus conference such that future systematic reviews and meta‐analyses can draw more robust and actionable conclusions.

## AUTHOR CONTRIBUTIONS

Amir A Jamali and A.P. designed the study, performed the literature search, wrote the main manuscript text and revised the manuscript. Adithya Shekhar, Kian Bagheri and Eric Kwok collected, analysed, and interpreted the data and wrote and revised the manuscript. Susan L. Stewart analysed the data and performed the statistical analysis.

## CONFLICT OF INTEREST STATEMENT

The authors declare no conflicts of interest.

## ETHICS STATEMENT

The study was conducted according to standard guidelines for meta‐analyses. As this was a review of previously published reports, no ethics approval or consent to participate was obtained.

## PROTOCOL AND REGISTRATION

The protocol was registered prospectively with PROSPERO, CRD42021218023.

## Data Availability

The data sets used and/or analysed during the current study will be available if the reader contacts the corresponding author. The reader will require direct access to the source manuscripts through their publishers.

## References

[1] Ayhan E . Intraarticular injections (corticosteroid, hyaluronic acid, platelet rich plasma) for the knee osteoarthritis. World J Orthop. 2014;5:351–361. 25035839 10.5312/wjo.v5.i3.351 PMC4095029

[2] Bagga H , Burkhardt D , Sambrook P , March L . Longterm effects of intraarticular hyaluronan on synovial fluid in osteoarthritis of the knee. J Rheumatol. 2006;33:946–950. 16652425

[3] Basnaev UI , Karakursakov NE , Mykhaylichenko VY , Kriventsov MA . Platelet‐rich plasma administering in osteoarthrosis treatment. Russian Open Med J. 2021;10:1–7.

[4] Belk JW , Kraeutler MJ , Houck DA , Goodrich JA , Dragoo JL , McCarty EC . Platelet‐rich plasma versus hyaluronic acid for knee osteoarthritis: a systematic review and meta‐analysis of randomized controlled trials. Am J Sports Med. 2020;49(1):249–260. 32302218 10.1177/0363546520909397

[5] Belk JW , Lim JJ , Keeter C , McCulloch PC , Houck DA , McCarty EC , et al. Patients with knee osteoarthritis who receive platelet‐rich plasma or bone marrow aspirate concentrate injections have better outcomes than patients who receive hyaluronic acid: systematic review and meta‐analysis. Arthroscopy. 2023;39:1714–1734. 36913992 10.1016/j.arthro.2023.03.001

[6] Buendía‐López D , Medina‐Quirós M , Fernández‐Villacañas marín MÁ . Clinical and radiographic comparison of a single LP‐PRP injection, a single hyaluronic acid injection and daily NSAID administration with a 52‐week follow‐up: a randomized controlled trial. J Orthop Traumatol. 2018;19:3. 30128934 10.1186/s10195-018-0501-3 PMC6102156

[7] Chen Z , Wang C , You D , Zhao S , Zhu Z , Xu M . Platelet‐rich plasma versus hyaluronic acid in the treatment of knee osteoarthritis: a meta‐analysis. Medicine. 2020;99:e19388. 32176063 10.1097/MD.0000000000019388 PMC7220139

[8] Cole BJ , Karas V , Hussey K , Merkow DB , Pilz K , Fortier LA . Hyaluronic acid versus platelet‐rich plasma: a prospective, double‐blind randomized controlled trial comparing clinical outcomes and effects on intra‐articular biology for the treatment of knee osteoarthritis. Am J Sports Med. 2017;45:339–346. 28146403 10.1177/0363546516665809

[9] DeLong JM , Russell RP , Mazzocca AD . Platelet‐rich plasma: the PAW classification system. Arthroscopy. 2012;28:998–1009. 22738751 10.1016/j.arthro.2012.04.148

[10] Di Martino A , Di Matteo B , Papio T , Tentoni F , Selleri F , Cenacchi A , et al. Platelet‐rich plasma versus hyaluronic acid injections for the treatment of knee osteoarthritis: results at 5 years of a double‐blind, randomized controlled trial. Am J Sports Med. 2019;47:347–354. 30545242 10.1177/0363546518814532

[11] Dulay GS , Cooper C , Dennison EM . Knee pain, knee injury, knee osteoarthritis & work, best practice & research. Clin Rheumatol. 2015;29:454–461. 10.1016/j.berh.2015.05.005 26612241

[12] Dulic O , Rasovic P , Lalic I , Kecojevic V , Gavrilovic G , Abazovic D , et al. Bone marrow aspirate concentrate versus platelet rich plasma or hyaluronic acid for the treatment of knee osteoarthritis. Medicina. 2021;57:1193. 34833411 10.3390/medicina57111193 PMC8623697

[13] Felson DT , Naimark A , Anderson J , Kazis L , Castelli W , Meenan RF . The prevalence of knee osteoarthritis in the elderly. The Framingham Osteoarthritis Study. Arthritis Rheumatism. 1987;30:914–918. 3632732 10.1002/art.1780300811

[14] Filardo G , Di Matteo B , Di Martino A , Merli ML , Cenacchi A , Fornasari P , et al. Platelet‐rich plasma intra‐articular knee injections show no superiority versus viscosupplementation: a randomized controlled trial. Am J Sports Med. 2015;43:1575–1582. 25952818 10.1177/0363546515582027

[15] Filardo G , Kon E , Di Martino A , Di Matteo B , Merli ML , Cenacchi A , et al. Platelet‐rich plasma vs hyaluronic acid to treat knee degenerative pathology: study design and preliminary results of a randomized controlled trial. BMC Musculoskelet Disord. 2012;13:229. 23176112 10.1186/1471-2474-13-229 PMC3532098

[16] Filardo G , Previtali D , Napoli F , Candrian C , Zaffagnini S , Grassi A . PRP injections for the treatment of knee osteoarthritis: a meta‐analysis of randomized controlled trials. Cartilage. 2021;13:364S–375S. 32551947 10.1177/1947603520931170 PMC8808870

[17] Goldberg VM , Buckwalter JA . Hyaluronans in the treatment of osteoarthritis of the knee: evidence for disease‐modifying activity. Osteoarthritis Cartilage. 2005;13:216–224. 15727888 10.1016/j.joca.2004.11.010

[18] Görmeli G , Görmeli CA , Ataoglu B , Çolak C , Aslantürk O , Ertem K . Multiple PRP injections are more effective than single injections and hyaluronic acid in knees with early osteoarthritis: a randomized, double‐blind, placebo‐controlled trial. Knee Surg Sports Traumatol Arthrosc. 2017;25:958–965. 26233594 10.1007/s00167-015-3705-6

[19] Guidolin DD , Ronchetti IP , Lini E , Guerra D , Frizziero L . Morphological analysis of articular cartilage biopsies from a randomized, clinical study comparing the effects of 500‐730 kDa sodium hyaluronate (Hyalgan) and methylprednisolone acetate on primary osteoarthritis of the knee. Osteoarthritis Cartilage. 2001;9:371–381. 11399102 10.1053/joca.2000.0398

[20] Herdman M , Gudex C , Lloyd A , Janssen M , Kind P , Parkin D , et al. Development and preliminary testing of the new five‐level version of EQ‐5D (EQ‐5D‐5L). Qual Life Res. 2011;20:1727–1736. 21479777 10.1007/s11136-011-9903-x PMC3220807

[21] Hohmann E , Tetsworth K , Glatt V . Is platelet‐rich plasma effective for the treatment of knee osteoarthritis? A systematic review and meta‐analysis of level 1 and 2 randomized controlled trials. Eur J Orthop Surg Traumatol. 2020;30:955–967. 32060630 10.1007/s00590-020-02623-4

[22] Jalali Jivan S , Monzavi SM , Zargaran B , Hamidi Alamdari D , Tavakol Afshari J , Etemad‐Rezaie A , et al. Comparative analysis of the effectiveness of intra‐articular injection of platelet‐rich plasma versus hyaluronic acid for knee osteoarthritis: results of an open‐label trial. Arch Bone Joint Surg. 2021;9:487–495. 34692930 10.22038/abjs.2021.52003.2569 PMC8503763

[23] Johnson JA , Pickard AS . Comparison of the EQ‐5D and SF‐12 health surveys in a general population survey in Alberta, Canada. Med Care. 2000;38:115–121. 10630726 10.1097/00005650-200001000-00013

[24] Kesiktas FN , Dernek B , Sen EI , Albayrak HN , Aydin T , Yildiz M . Comparison of the short‐term results of single‐dose intra‐articular peptide with hyaluronic acid and platelet‐rich plasma injections in knee osteoarthritis: a randomized study. Clin Rheumatol. 2020;39:3057–3064. 32358661 10.1007/s10067-020-05121-4 PMC7497346

[25] Küçükakkaş O , Aydin T , Yurdakul OV . Evaluation of the effect of intra‐articular platelet‐rich plasma and hyaluronic acid injections on femoral cartilage thickness in chronic knee osteoarthritis. Acta Orthop Belg. 2022;88:811–819. 36800668 10.52628/88.4.10243

[26] Lana JF , Weglein A , Sampson SE , Vicente EF , Huber SC , Souza CV , et al. Randomized controlled trial comparing hyaluronic acid, platelet‐rich plasma and the combination of both in the treatment of mild and moderate osteoarthritis of the knee. J Stem Cells Regener Med. 2016;12:69–78. 10.46582/jsrm.1202011 PMC5227106 28096631

[27] Lawrence RC , Felson DT , Helmick CG , Arnold LM , Choi H , Deyo RA , et al. Estimates of the prevalence of arthritis and other rheumatic conditions in the United States. Part II. Arthritis Rheumatism. 2008;58:26–35. 18163497 10.1002/art.23176 PMC3266664

[28] Lin KY , Yang CC , Hsu CJ , Yeh ML , Renn JH . Intra‐articular injection of platelet‐rich plasma is superior to hyaluronic acid or saline solution in the treatment of mild to moderate knee osteoarthritis: a randomized, double‐blind, triple‐parallel, placebo‐controlled clinical trial. Arthroscopy. 2019;35:106–117. 30611335 10.1016/j.arthro.2018.06.035

[29] Lisi C , Perotti C , Scudeller L , Sammarchi L , Dametti F , Musella V , et al. Treatment of knee osteoarthritis: platelet‐derived growth factors vs. hyaluronic acid. A randomized controlled trial. Clin Rehabil. 2018;32:330–339. 28783969 10.1177/0269215517724193

[30] Listrat V , Ayral X , Patarnello F , Bonvarlet JP , Simonnet J , Amor B , et al. Arthroscopic evaluation of potential structure modifying activity of hyaluronan (Hyalgan) in osteoarthritis of the knee. Osteoarthritis Cartilage. 1997;5:153–160. 9219678 10.1016/s1063-4584(97)80010-6

[31] McCaffrey N , Kaambwa B , Currow DC , Ratcliffe J . Health‐related quality of life measured using the EQ‐5D‐5L: South Australian population norms. Health Qual Life Outcomes. 2016;14:133. 27644755 10.1186/s12955-016-0537-0 PMC5028927

[32] Montañez‐Heredia E , Irízar S , Huertas P , Otero E , Del Valle M , Prat I , et al. Intra‐articular injections of platelet‐rich plasma versus hyaluronic acid in the treatment of osteoarthritic knee pain: A randomized clinical trial in the context of the Spanish national health care system. Int J Mol Sci. 2016;17:1064. 27384560 10.3390/ijms17071064 PMC4964440

[33] Moreland LW . Intra‐articular hyaluronan (hyaluronic acid) and hylans for the treatment of osteoarthritis: mechanisms of action. Arthritis Res Ther. 2003;5:54–67. 12718745 10.1186/ar623 PMC165033

[34] Pasquali Ronchetti I , Guerra D , Taparelli F , Boraldi F , Bergamini G , Mori G , et al. Morphological analysis of knee synovial membrane biopsies from a randomized controlled clinical study comparing the effects of sodium hyaluronate (Hyalgan) and methylprednisolone acetate (Depomedrol) in osteoarthritis. Rheumatology. 2001;40:158–169. 11257152 10.1093/rheumatology/40.2.158

[35] Raeissadat SA , Ghazi Hosseini P , Bahrami MH , Salman Roghani R , Fathi M , Gharooee Ahangar A , et al. The comparison effects of intra‐articular injection of Platelet Rich Plasma (PRP), Plasma Rich in Growth Factor (PRGF), Hyaluronic Acid (HA), and ozone in knee osteoarthritis; a one year randomized clinical trial. BMC Musculoskelet Disord. 2021;22:134. 33536010 10.1186/s12891-021-04017-x PMC7860007

[36] Raeissadat SA , Ghorbani E , Sanei Taheri M , Soleimani R , Rayegani SM , Babaee M , et al. MRI changes after platelet rich plasma injection in knee osteoarthritis (randomized clinical trial). J Pain Res. 2020;13:65–73. 32021396 10.2147/JPR.S204788 PMC6959502

[37] Sdeek M , Sabry D , El‐Sdeek H , Darweash A . Intra‐articular injection of platelet rich plasma versus hyaluronic acid for moderate knee osteoarthritis. A prospective, double‐blind randomized controlled trial on 189 patients with follow‐up for three years. Acta Orthop Belg. 2021;87:729–734. 35172440 10.52628/87.4.18

[38] Spaková T , Rosocha J , Lacko M , Harvanová D , Gharaibeh A . Treatment of knee joint osteoarthritis with autologous platelet‐rich plasma in comparison with hyaluronic acid. Am J Phys Med Rehabil. 2012;91:411–417. 22513879 10.1097/PHM.0b013e3182aab72

[39] Sterne JAC , Savović J , Page MJ , Elbers RG , Blencowe NS , Boutron I , et al. RoB 2: a revised tool for assessing risk of bias in randomised trials. BMJ. 2019;1:l4898. 10.1136/bmj.l4898 31462531

[40] Su K , Bai Y , Wang J , Zhang H , Liu H , Ma S . Comparison of hyaluronic acid and PRP intra‐articular injection with combined intra‐articular and intraosseous PRP injections to treat patients with knee osteoarthritis. Clin Rheumatol. 2018;37:1341–1350. 29388085 10.1007/s10067-018-3985-6

[41] Tan J , Chen H , Zhao L , Huang W . Platelet‐rich plasma versus hyaluronic acid in the treatment of knee osteoarthritis: a meta‐analysis of 26 randomized controlled trials. Arthroscopy. 2021;37:309–325. 32679294 10.1016/j.arthro.2020.07.011

[42] Tang JZ , Nie MJ , Zhao JZ , Zhang GC , Zhang Q , Wang B . Platelet‐rich plasma versus hyaluronic acid in the treatment of knee osteoarthritis: a meta‐analysis. J Orthop Surg. 2020;15:403. 10.1186/s13018-020-01919-9 PMC7488405 32912243

[43] Tavassoli M , Janmohammadi N , Hosseini A , Khafri S , Esmaeilnejad‐Ganji SM . Single‐ and double‐dose of platelet‐rich plasma versus hyaluronic acid for treatment of knee osteoarthritis: a randomized controlled trial. World J Orthop. 2019;10:310–326. 31572668 10.5312/wjo.v10.i9.310 PMC6766465

[44] Tschopp M , Pfirrmann CWA , Fucentese SF , Brunner F , Catanzaro S , Kühne N , et al. A randomized trial of intra‐articular injection therapy for knee osteoarthritis. Invest Radiol. 2023;58:355–362. 36728848 10.1097/RLI.0000000000000942 PMC10090303

[45] Vaquerizo V , Plasencia MÁ , Arribas I , Seijas R , Padilla S , Orive G , et al. Comparison of intra‐articular injections of plasma rich in growth factors (PRGF‐Endoret) versus Durolane hyaluronic acid in the treatment of patients with symptomatic osteoarthritis: a randomized controlled trial. Arthroscopy. 2013;29:1635–1643. 24075613 10.1016/j.arthro.2013.07.264

[46] Wang Y , Hall S , Hanna F , Wluka AE , Grant G , Marks P , et al. Effects of Hylan G‐F 20 supplementation on cartilage preservation detected by magnetic resonance imaging in osteoarthritis of the knee: a two‐year single‐blind clinical trial. BMC Musculoskelet Disord. 2011;12:195. 21861935 10.1186/1471-2474-12-195 PMC3201041

[47] Wang YC , Lee CL , Chen YJ , Tien YC , Lin SY , Chen CH , et al. Comparing the efficacy of intra‐articular single platelet‐rich plasma (PRP) versus novel crosslinked hyaluronic acid for early‐stage knee osteoarthritis: a prospective, double‐blind, randomized controlled trial. Medicina. 2022;58:1028. 36013495 10.3390/medicina58081028 PMC9415551

[48] Yaradilmis YU , Demirkale I , Safa Tagral A , Caner Okkaoglu M , Ates A , Altay M . Comparison of two platelet rich plasma formulations with viscosupplementation in treatment of moderate grade gonarthrosis: a prospective randomized controlled study. J Orthop. 2020;20:240–246. 32071523 10.1016/j.jor.2020.01.041 PMC7011002

[49] Yu W , Xu P , Huang G , Liu L . Clinical therapy of hyaluronic acid combined with platelet‐rich plasma for the treatment of knee osteoarthritis. Exp Ther Med. 2018;16:2119–2125. 30186448 10.3892/etm.2018.6412 PMC6122407

